# Genetic and molecular dynamics analysis of two variants of the *COL4A5* gene causing Alport syndrome

**DOI:** 10.1186/s12920-023-01623-7

**Published:** 2023-08-18

**Authors:** Lei Liang, Haotian Wu, Zeyu Cai, Jianrong Zhao

**Affiliations:** 1 Center for Prenatal Diagnosis and Medical Genetics, Affiliated Hospital of Inner Mongolia Medical University, Hohhot, 010015 PR China; 2 School of Public Health, Inner Mongolia Medical University, Hohhot, 010015 PR China; 3 Department of Nephrology, Affiliated Hospital of Inner Mongolia Medical University, Hohhot, 010015 PR China

**Keywords:** *COL4A5* gene, Alport syndrome, Minigene assay, Splicing variant, Molecular dynamics

## Abstract

**Background:**

Alport syndrome (AS; OMIM#308,940) is a hereditary kidney disease that progresses over time and is distinguished by hearing loss and ocular irregularities. The syndrome has three subtypes, namely X-linked (XL; OMIM#301,050), autosomal recessive (AR; OMIM#203,780), and autosomal dominant (AD; OMIM#104,200), which are categorized based on their respective modes of inheritance. XLAS is attributed to a pathogenic variant in the *COL4A5* (OMIM*303,630) gene, which encodes the α5(IV) chain of type IV collagen (Col-IV). In contrast, ADAS and ARAS are the result of variants in the *COL4A3* (OMIM*120,070) and *COL4A4* (OMIM*120,131) genes, which encode the α3(IV) and α4(IV) chains of Col-IV, respectively. Typically, the diagnosis of AS necessitates hereditary or pathological assessments. The determination of splicing variants as pathogenic or non-pathogenic based on gene sequencing outcomes is challenging.

**Methods:**

In this study, we conducted exome sequencing and Sanger sequencing on two unrelated Chinese patients with AS. We identified a deletion variant c.4414delG in the *COL4A5* gene and a splicing variant c.4298-20T > A in the same gene. In order to ascertain the impact of c.4298-20T > A on the synthesis of *COL4A5* mRNA, we performed experiments involving minigene splicing. Additionally, we predicted the ability of these two variants to affect triple helix formation of α345(IV) using molecular dynamics methods.

**Results:**

The c.4414delG deletion variant caused a change in the genetic code of the *COL4A5* gene. Specifically, it caused a shift in codon 1472 from encoding aspartate to encoding methionine. This shift resulted in a change of 75 amino acids in the protein sequence, ultimately leading to an early stop codon. This premature stop codon caused the production of a truncated α5(IV) chain with a predicted protein effect of p.D1472Mfs. The mRNA of the *COL4A5* gene experienced intron 46 retention due to the splicing variant c.4298-20T > A, leading to the inclusion of six additional amino acids between amino acids 1432 and 1433 of the α5(IV) chain. This variant is predicted to have a protein effect of p.(P1432_G1433insDYFVEI). The impact of two variants, c.4414delG and c.4298-20T > A, on the aggregation region for α3(IV), α4(IV), and α5(IV) trimerisation were studied using molecular dynamics simulations. Results showed that the deletion variant c.4414delG had a significantly stronger disruption on NC1, compared to the splicing variant c.4298-20T > A. This difference in impact is consistent with the varying clinical phenotypes observed in the two patients. Based on the American College of Medical Genetics and Genomics (ACMG) classification criteria and guidelines for genetic variants, the deletion variant c.4414delG was rated as pathogenic while the splicing variant c.4298-20T > A was rated as likely-pathogenic.

**Conclusion:**

Our study has identified two novel pathogenic loci, the deletion variant c.4414delG and the splicing variant c.4298-20T > A, associated with XLAS. This finding expands the genetic spectrum of XLAS. We suggest that molecular dynamics can effectively model the effect of genetic variation on α345(IV) trimerization, which may offer valuable insights into the mechanisms of XLAS pathogenesis.

**Supplementary Information:**

The online version contains supplementary material available at 10.1186/s12920-023-01623-7.

## Introduction

Alport syndrome (AS) is an inherited condition characterized by a gradual decline in renal function, sensorineural hearing loss, and ocular anomalies [1, 2]. *COL4A3*, *COL4A4*, and *COL4A5* are responsible for encoding the α3(IV), α4(IV), and α5(IV) chains of type IV collagen (Col-IV), respectively. AS arises from a genetic anomaly in one of the three chains. The condition is classified into three subtypes, namely XLAS, ARAS and ADAS, based on the pattern of inheritance. XLAS is the most prevalent type of AS, responsible for 80% of all AS cases. It is caused by a mutation in the *COL4A5* gene which encodes α5(IV) [3–5]. XLAS patients have been found to exhibit a significant number of variants, with missense variants being the most prevalent (approximately 38.0%), followed by deletion variants (estimated 15.9%) and splice variants (approximately 14.9%) [6]. This study identified two different variants in the *COL4A5* gene, a deletion variant (c.4414delG) and a splice variant (c.4298-20T > A), in two unrelated Chinese patients with XLAS. The study then assessed the severity of these variants in disrupting the trimerization of Col-IV. Furthermore, the study introduces a unique concept of using molecular dynamics principles to predict the impact of *COL4A5* variants on Col-IV trimerization.

## Materials and methods

### Ethical compliance and patient information

Patient 1: A 23-year-old male patient underwent a renal puncture due to proteinuria that persisted for two weeks. Patho-logical examination confirmed a diagnosis of Alport syndrome. The patient also suffered from significant hearing and vision loss, leading to genetic testing in 2021. Nonetheless, manual microscopy for urine sedimentation or genetic testing was not performed on the patient’s parents and 5-year-old brother due to their manifestation of normal phenotypes. As of 2022, the patient continues to experience persistent hearing loss. He has also undergone IOL replacement and are now beginning peritoneal dialysis to manage his end-stage renal disease (ESRD). The patient’s kidney function was severely impaired prior to beginning peritoneal dialysis. The results of the patient’s blood tests showed elevated levels of urea at 18.3mmol/L and creatinine at 1237mmol/L. The pa-tient’s haemoglobin level was 143 g/L, blood potassium was 4.70 mmol/L, and blood calcium was 2.02mmol/L. In addition to these symptoms, the patient also experienced extra-renal symptoms, including loss of vision in both eyes and sensorineural hearing loss in both ears.

Patient 2: A male patient, aged 15, was hospitalized for a period of two weeks due to urinary protein. The patient had no known familial history of renal disease, and both parents exhibited normal phenotypes. Upon examination, the patient’s abdomen was found to be soft and non-tender, with no palpable liver or spleen enlargement. Additionally, there was no percussion pain in either kidney area, nor was there any edema present in the lower extremities. Auxiliary examination: the results of the urinalysis revealed a urine protein level of 2+, occult blood level of 2+, urine creatinine level of 7897µmol/L, and a 24-hour urine protein quantification of 6.76 g/24 h. The patient exhibited normal hearing function upon undergoing an auditory test and did not display any ocular abnormalities during examination by an ophthalmologist. The renal electron microscopy findings exhibited irregular thinning and thickening, along with a diffuse basket-weave pattern that strongly indicated the presence of AS.

The Ethics Committee of Inner Mongolia Medical University granted approval for this study, and written informed consent was obtained from the patients.

### Whole exome sequencing and sanger sequencing

Peripheral blood samples were obtained from patients using EDTA anticoagulation tubes. The extraction of genomic DNA was performed utilizing the Blood Genomic DNA Extraction Kit (TIANGEN, China). The Hieff NGS®OnePot DNA Library Prep Kit for Illumina® (YEASEN, China) and xGen Exome Research Panel v1.0 (Integrated DNA Technologies, Inc., United States) were utilized for the library preparation and exome capture, respectively. The captured libraries were subsequently subjected to sequencing on a HiSeq2500 (Illumina, California, CA, USA) platform.

The raw data underwent quality assessment via FASTQC, followed by mapping of clean reads to the reference genome (GRCh37/hg19) utilizing BWA software. Subsequently, the GATK pipeline was employed to call SNP and Indel variants, with duplicate removal and base quality recalibration performed beforehand. The variant annotation process was conducted using ANNOVAR, whereby filtering criteria were applied based on minor allele frequencies (MAFs) of less than 0.5% for the dbSNP, 1000G, ExAC, and gnomAD databases.

The guidelines for interpreting sequence variation data pertain to the genetic variation classification standards and guidelines of the American College of Medical Genetics and Genomics (ACMG). To verify the authenticity of the variants, two patients’ peripheral blood DNA samples were subjected to Sanger sequencing. The resulting amplicon was sequenced using the ABI 3730xl Genetic Analyzer (Applied Biosystems, Inc.), and the sequences were compared to reference sequences using CodonCode Aligner.

### Construction of in Vitro expression vector

The construction of wild-type and mutant type plasmids was based on the mutation *COL4A5*: c.4298-20T > A, utilizing the pMini-CopGFP cloning vector with BamHI/XhoI as the clone site. Seamless primers were utilized to amplify both normal genomic DNA and genomic DNA containing the c.4298-20T > A mutation. The fragments of the wild-type and mutant target genes were acquired and integrated into the cloning vector through a dual enzyme digestion and recombination process. The resulting recombinant product was subsequently transformed into proficient cells and cultured, with clones being chosen for PCR amplification. Sanger sequencing was then conducted to ascertain the accuracy of the insertion of the COL4A5 gene’s target fragment into the vector. The appropriate recombinant plasmids for the wild-type and mutant minigenes were selected.

### RT-PCR, PCR and sequencing

Following the cultivation of a bacterial solution of 5-15ml, the plasmid was extracted and subsequently transfected into 293T cells. Subsequent to transfection, cDNA was extracted from the cells via RNA reverse transcription, followed by the design of primers intended for the amplification of RT-PCR and subsequent gel electrophoresis.

### The evolutionary conservation analysis of amino acid residues of mutant proteins

The protein sequences of various species were obtained from the National Center for Biotechnology Information (NCBI) and subjected to multiple sequence alignment and conservation analysis using the Jalview software.

### 3-D structure analysis of α5(IV) and α345(IV)

The homology modeling technique was employed using MODELLER software to generate the 3-D structure of both the wild-α345(IV) trimer and monomer. The selection of PDB IDs 3HQV, 5NB0, 5NB1, and 5NAZ as templates for the triple helical region and the NC1 domains of α3(IV)–α5(IV) facilitated the process. The 3-D structures of p.D1472Mfs, p.(1432_G1433insDYFVEI) and their mutant trimers were prepared from the amino acid sequences of wild-α5(IV). The resulting structures were subjected to structural optimization by GROMACS version 2020.4 (http://manual.gromacs.org). The AMBER14SB force field and TIP3P water model were used for all simulations. The protein structures were visualized via VMD software (https://www.ks.uiuc.edu/Research/vmd).

### Molecular dynamics

The protein molecular dynamics were simulated using the GROMACS software package, employing the AMBER14sb force field. The protein was incorporated into the GROMACS module, and supplemented with hydrogen atoms and NaCl ions. The TIP3P dominant water model was selected, and periodic boundary conditions were established. The molecular dynamics simulation workflow encompassed four stages: energy minimization, NVT equilibrium, NPT equilibrium, and production dynamics simulation. Initially, the heavy atoms of the protein were constrained to reduce the energy of water molecules through a process of 10,000 steps, which involved both the steepest descent method and the conjugate gradient method (5,000 steps each). Subsequently, the constraints were upheld, and a simulation of the entire system was conducted using the NVT ensemble for 50,000 steps. The experiment involved conducting a 50,000 step NPT ensemble simulation of the entire system at a temperature of 298 K and a time step of 2 fs. A molecular dynamics simulation of the system was subsequently conducted in the NPT ensemble, utilizing a time step of 2 fs and lasting for a duration of 100ns. The pertinent parameters were scrutinized utilizing the GROMACS software package module.

## Results

### Pathologic diagnosis

**Fig. 1:**
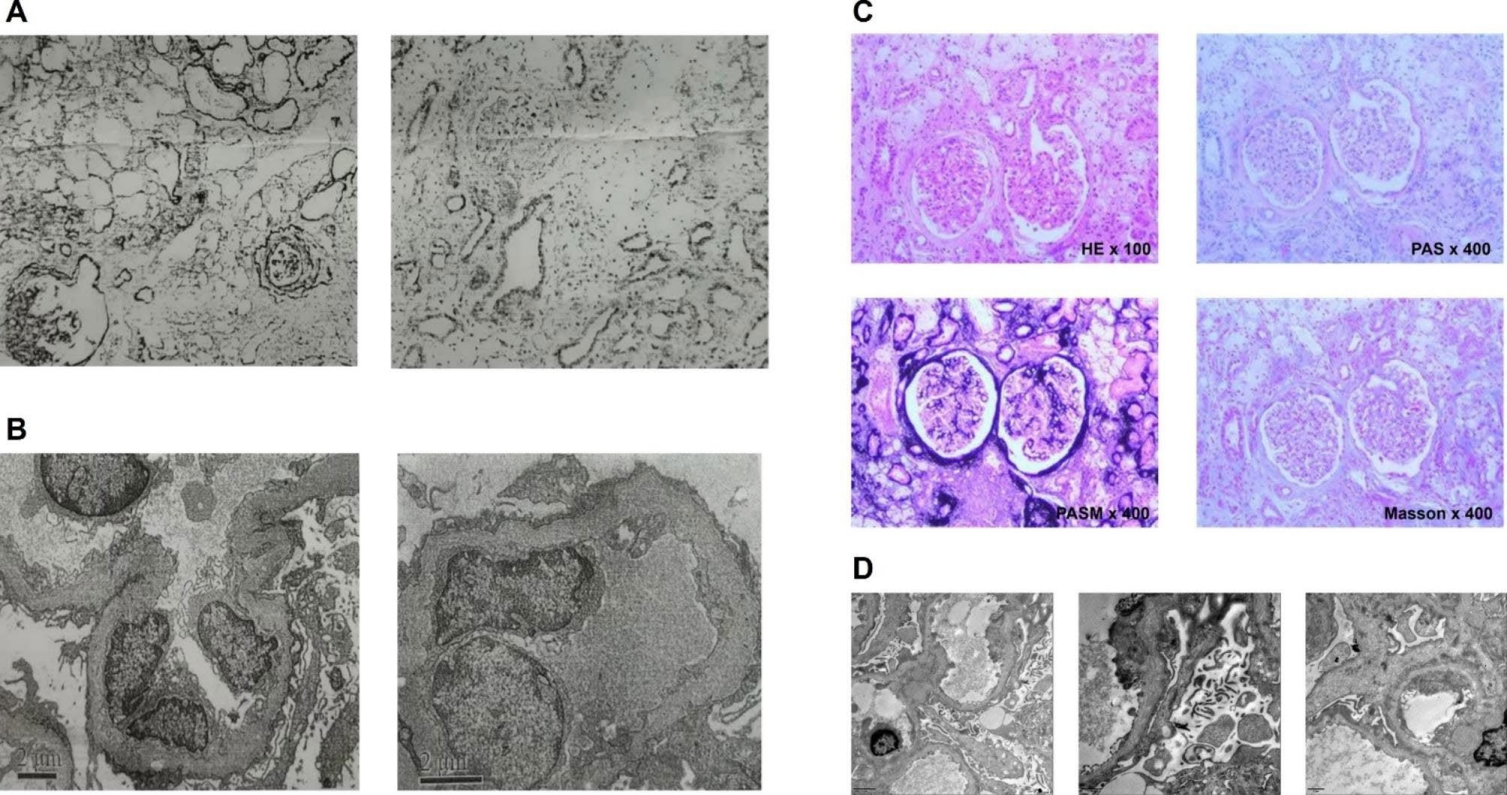
The pathology of renal tissue biopsy in patients 1 and 2. (**A**) The kidney tissue obtained from patient 1 was subjected to immunofluorescence analysis. Upon examination with a light microscope, renal puncture tissue revealed the presence of 16 glomeruli. Of those, two cases exhibited globular sclerosis, accompanied by moderate proliferation of glomerular Mesangial cells and matrix. Additionally, irregular thickening of the basement membrane was observed, along with the formation of a fibrous crescent in two cells and segmental sclerosis in four segments. The renal tubular epithelium has undergone severe vacuolar degeneration, resulting in multifocal and patchy atrophy. Additionally, the renal interstitial area shows multifocal and patchy infiltration of lymphoid cells, monocytes, and foam cells, accompanied by fibrosis. Arteriole walls have thickened, and there is intimal hyperplasia, leading to lumen stenosis. (**B**) The ultrastructural analysis of the kidney tissue obtained from patient (1) The electron microscope revealed mild to moderate proliferation of glomerular mesangial cells and matrix, irregular thickening of the basement membrane, strat-ified dense layer and insect-like changes. There was extensive fusion of epithelial foot processes and no electron dense deposition. Additionally, there was renal tubular epithelial vacuolar degeneration, partial atrophy, renal interstitial lymphoid monocytes and foam cell infiltration with collagen fiber proliferation. (**C**) Histological examination was performed on the kidney of patient (2) The individual exhibited granular degeneration in the renal tubular epithelial cells, accompanied by focal foam-like cell infiltration. The Periodic Acid-Schiff staining revealed sclerotic glomeruli and foam-like cell infiltration in close proximity to the sclerosed glomeruli. Additionally, the Periodic Acid-Silver Methenamine staining displayed heterogeneous staining of the capillary loop basement membrane. However, the Masson staining did not indicate any discernible deposition of the furophilic protein in the glomeruli. (**D**) The kidney tissue obtained from patient 2 was subjected to ultrastructural analysis. The electron microscope images revealed the presence of cobweb-like structures and a torn basement membrane in all three samples

Patient 1: During the immunofluorescence examination, granular deposits were discovered in the mesangial region of the glomeruli, as well as tubular granular deposits in the renal tubular epithelium. Following light microscopy, the diagnosis of focal proliferative sclerosing glomerulopathy with IgA accumulation was made, but electron microscopy was still needed to rule out Alport syndrome (Fig. 1A). Ultimately, electron microscopy confirmed the diagnosis of Alport syndrome (Fig. 1B).

Patient 2: The tissue was procured through a renal puncture, followed by a standard renal pathological analysis. Hematoxylin and eosin staining revealed the presence of 10 glomeruli, of which 2 exhibited glomerular sclerosis. Additionally, 6 glomeruli were visualized through periodic acid-Schiff, periodic acid-Schiff with methenamine silver, and Masson trichrome staining, with 1 exhibiting glomerular sclerosis. Notably, there was no discernible endothelial cell proliferation or furoglobin deposition in the mesangial region. The capillary loop exhibited an open configuration, while the basement membrane displayed a slight thickening (Fig. 1C). The basement membrane exhibited a thickness ranging from 200 to 600 nm. The segmental basement membrane’s dense layer was observed to be thickened, with some instances of tearing and the manifestation of cobweb-like structures (Fig. 1D).

### Identification of *COL4A5* mutation

**Fig. 2:**
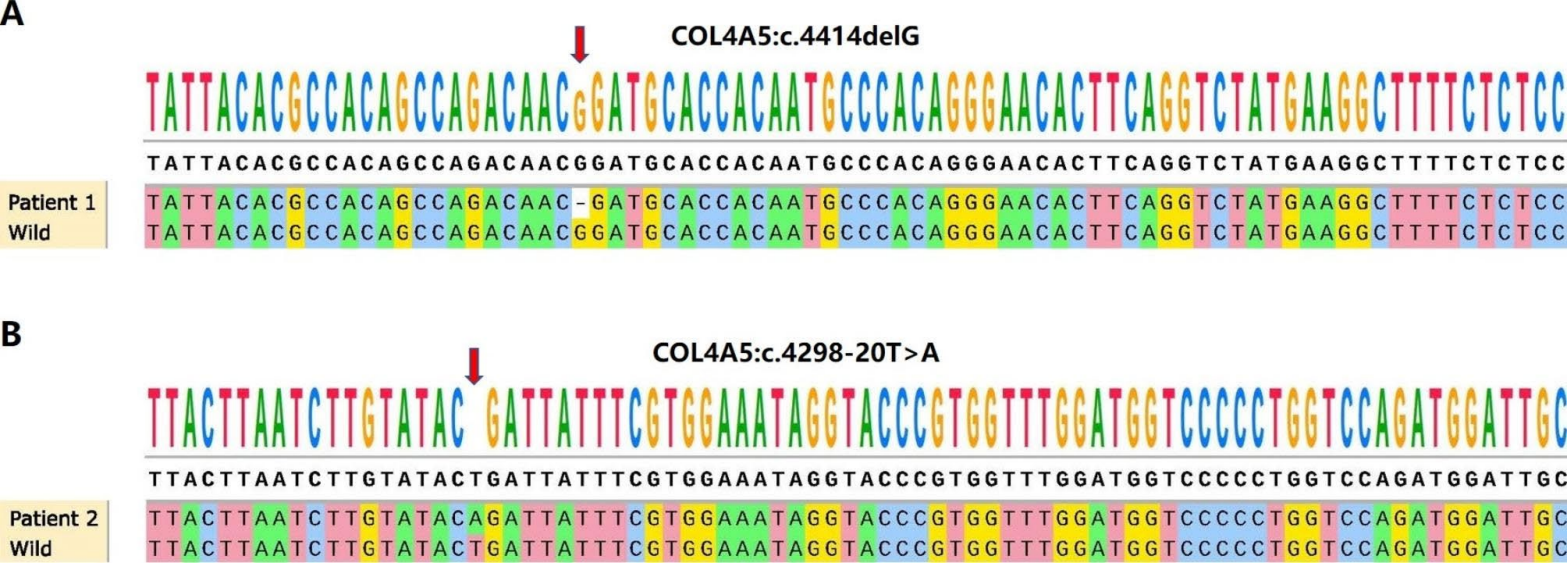
The genetic sequencing outcomes of two Chinese patients diagnosed with AS, who are not biologically related, were analyzed. (**A**) The gene sequencing results of patient 1. The utilization of WES and Sanger sequencing has led to the identification of a novel deletion variant, c.4414delG, located in exon 47 of the *COL4A5* gene in patient (1) The present variation has been indicated by a red arrow. (**B**) The gene sequencing results of patient (2) According to the bioinformatics software, dbscSNV and SpliceAI, it is predicted that this variant may lead to anomalous splicing of the *COL4A5* gene. The present variation has been indicated by a red arrow

The utilization of WES and Sanger sequencing has led to the identification of a novel deletion variant, c.4414delG, located in exon 47 of the COL4A5 gene in patient 1 (Fig. 2A). This deletion variant has caused a change in the *COL4A5* gene cDNA codon 1472, where it now encodes methionine instead of aspartic acid. This change ultimately results in a truncated coding sequence (p.D1472Mfs) due to the generation of an in-transit stop codon, which occurs after the protein has shifted by 75 amino acids.

Patient 2 was found to have the c.4298-20T > A variant in the *COL4A5* gene (Fig. 2B). The bioinformatics software, namely dbscSNV and SpliceAI, were utilized to conduct an analysis of c.4298-20T > A. According to the bioinformatics software outcomes, it is anticipated that the variant could potentially result in anomalous splicing of the COL4A5 mRNA.

### Corroboration of *COL4A5* mutations as the determinant of alternative splicing in splicing assay

**Fig. 3:**
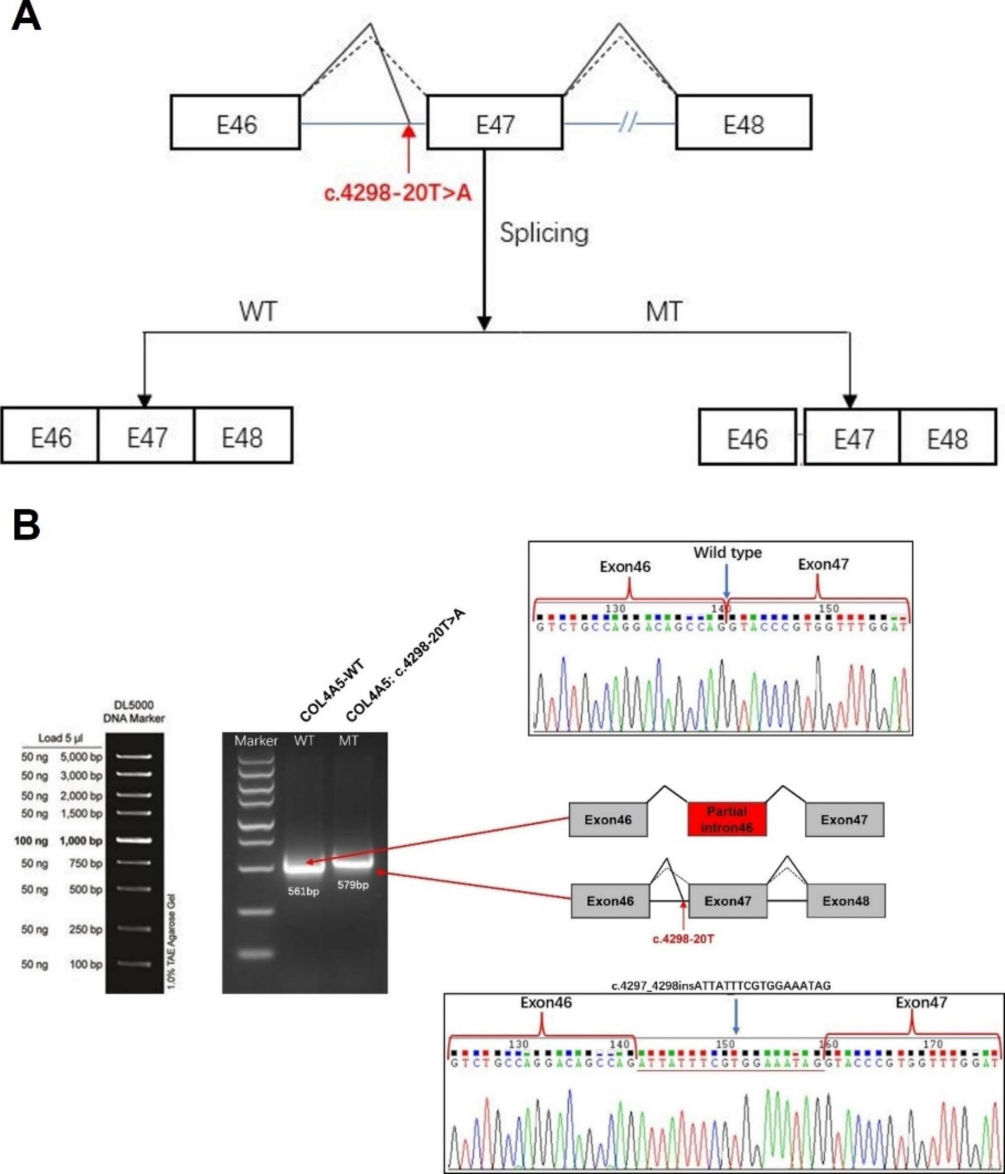
The splicing pattern of patient 2 was investigated using the *COL4A5* gene constructs. (**A**) A schematic diagram depicting splicing. The splicing schematic diagram indicates that the mutation in question may result in a splicing variation of exon 46 of the *COL4A5* gene, as predicted by the Bioinformatics software, dbscSNV and SpliceAI. (**B**) A splicing pattern of the *COL4A5* gene variant c.4298-20T > A was verified through a minigene experiment, wherein both the wild-type (*COL4A5*-WT) and mutant (*COL4A5*-MT) versions of the gene were introduced into 293T cells on a transient basis. Following the extraction of RNA, the splicing products underwent analysis via RT-PCR. The lower bands indicate accurate splicing, whereas the higher bands signify the insertion of 18 nucleotides in intron 46 of *COL4A5*

The plasmids targeting COL4A5, specifically the c.4298-20T > A mutation, were constructed for both the wild-type (WT) and mutant (MT) variants. These plasmids were subsequently transfected into 293T cells (Fig. 3A). Subsequently, RNA was isolated for reverse transcription, followed by PCR amplification of cDNA. The identification of abnormal splicing of the mutant mRNA was accomplished by analyzing the band size of PCR amplification products and Sanger sequencing outcomes. The findings revealed that the transcribed mRNA sequence of the wild-type plasmid was consistent with expectations, encompassing a complete mRNA product transcribed by exon46 and exon47. Conversely, the mutant plasmid’s sequence indicated the retention of 18 bp of intron46 and the impact of c.4298-20T > A (c.4297_4298insATTATTTCGTGGAAATAG) on mRNA splicing (Fig. 3B).

### Evolutionary conservation analysis of aberrant amino acid residues caused by *COL4A5* gene variants across species

**Fig. 4:**
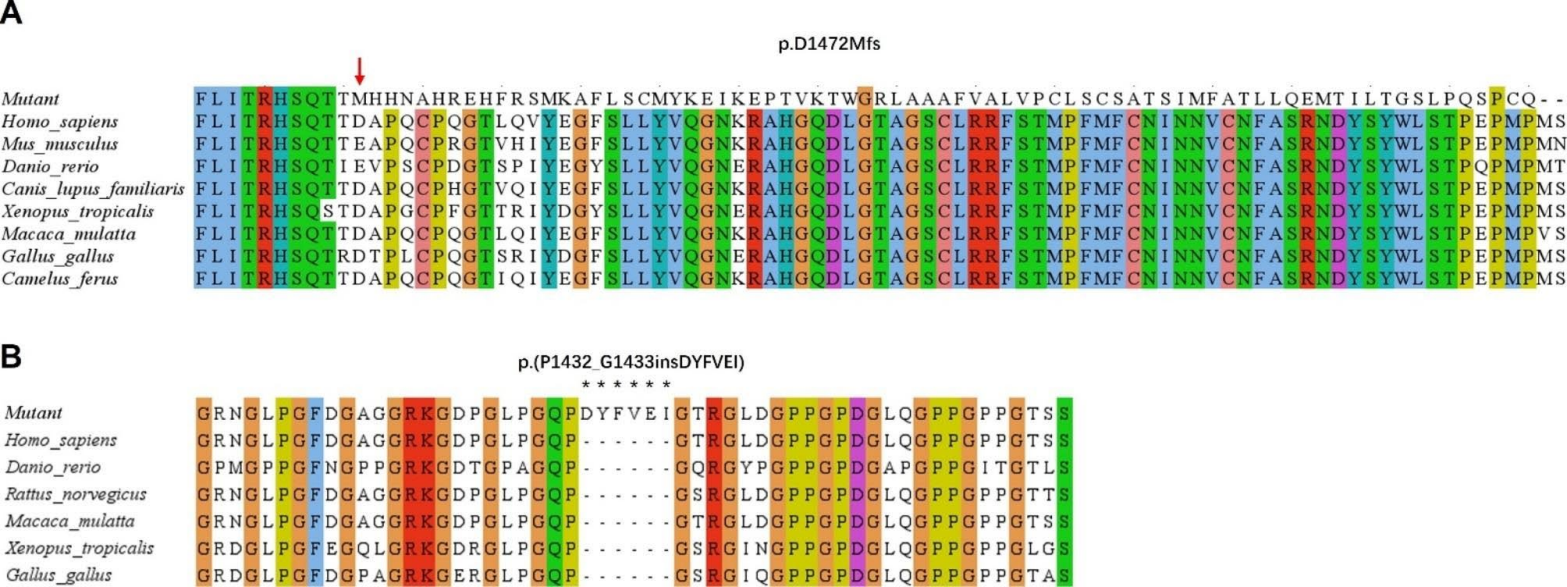
Evolutionary conservation analysis of aberrant amino acid residues caused by *COL4A5* gene variants across species. (**A**) The evolutionary conservation of amino acid residues that have been altered by p.D1472Mfs has been observed across various species. The NCBI accession numbers for these species are as follows: *Homo sapiens* (NP_000486.1), *Mus musculus* (001156627.1), *Danio rerio* (NP_001116702.1), *Canis lupus familiaris* (NP_001002979.1), *Xenopus tropicalis* (XP_004916922.2), *Macaca mulatta* (XP_014983488.2), and *Gallus gallus* (XP_015134092.2). The location of the code shift variation is indicated by a red arrow. (**B**) The present study examines the evolutionary preservation of amino acid residues that have been modified by the insertion of DYFVEI at position P1432_G1433 across various species. The NCBI accession numbers for the respective species are as follows: *Homo sapiens* (NP_000486.1), *Danio rerio* (NP_001116702.1), *Macaca mulatta* (XP_014983488.2), *Xenopus tropicalis* (XP_004916922.2), and *Gallus gallus* (XP_015134092.2). The asterisk (*) denotes the presence of inserted amino acids

The variant c.4414delG in *COL4A5* causes a frameshift and premature termination of the encoded protein at the aspartic acid position 1472. This leads to the production of a truncated protein, p.D1472Mfs, consisting of 1546 amino acids. Further analysis revealed that the affected amino acids were conserved across multiple species, indicating their importance in the protein’s function (Fig. 4A). The variant c.4298-20T > A in the *COL4A5* gene caused the protein it encodes to have six extra amino acids between P1432 and G1433, resulting in the amino acid sequence p.(P1432_G1433insDYFVEI). An analysis of evolutionary conservatism revealed that these additional amino acids are not present in several species (Fig. 4B).

### 3-D structure analysis of the α5(IV) and α345(IV) trimer

**Fig. 5:**
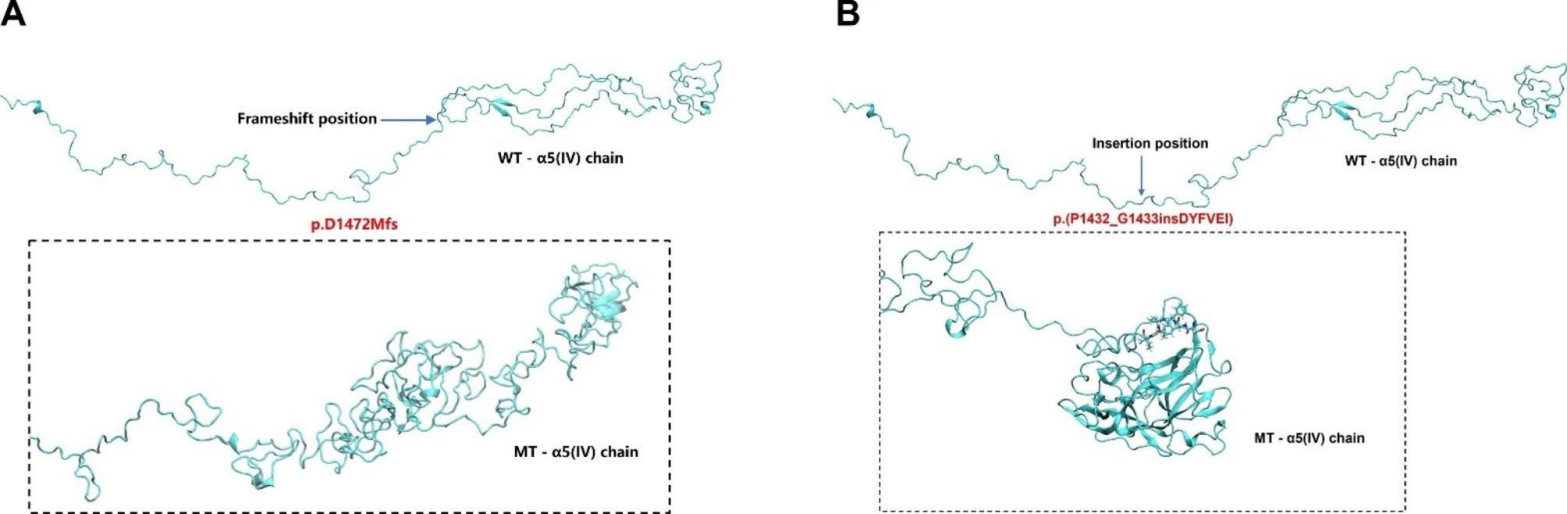
The molecular dynamics simulation lasted for 100 ns to obtain local magnification diagrams of the conformations of wild-α5(IV), mute1-α5(IV), and mute2-α5(IV) chains. (**A**) Enlarged view of residues after amino acid 1472 of α5(IV) following molecular dynamics simulations. (**B**) The blue label serves as a means of magnification to verify the presence of amino acids inserted at positions 1432–1433

**Fig. 6:**
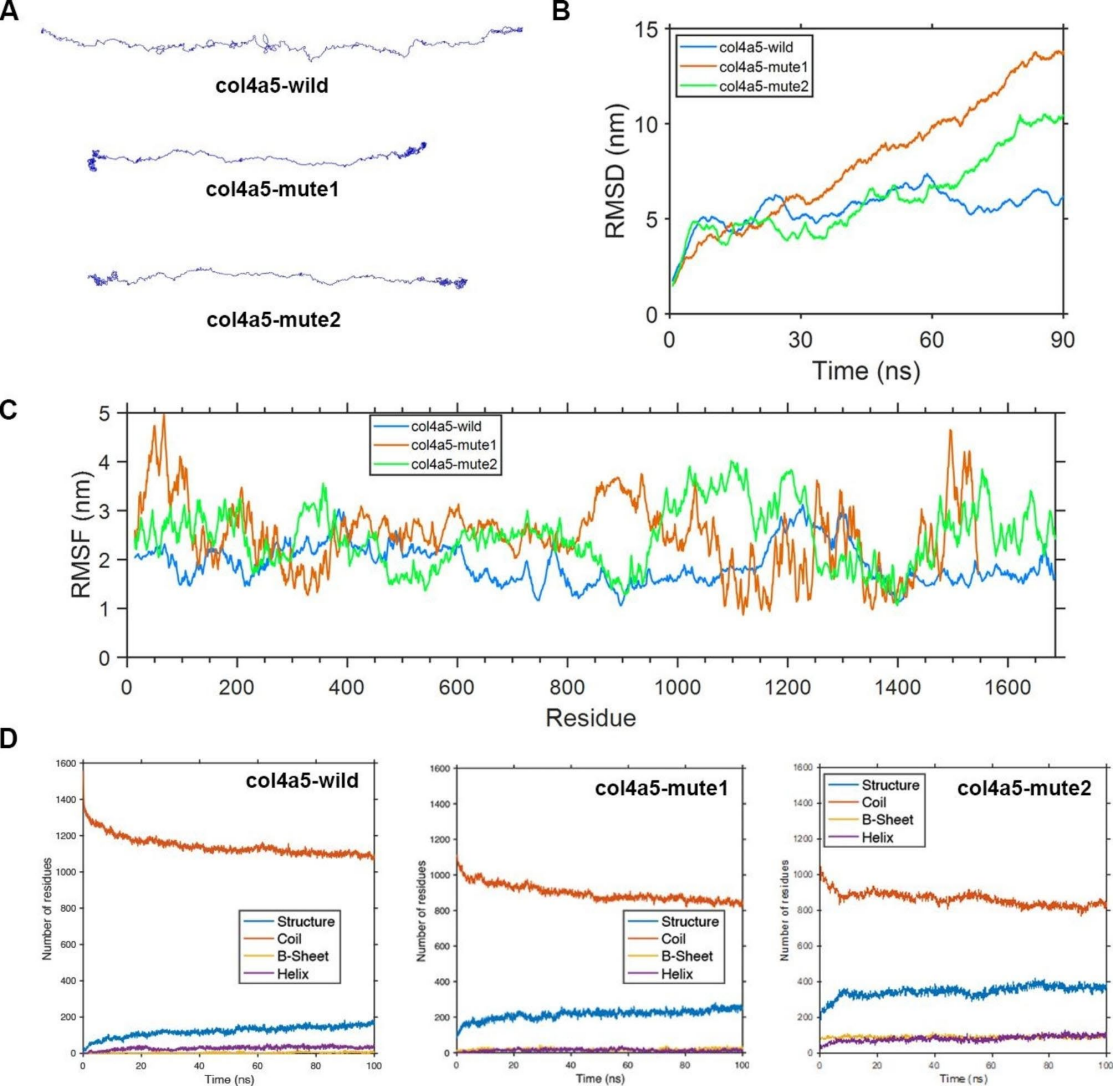
Protein molecular dynamics simulation of the α5(IV). (**A**) The conformation of the wild-type α5(IV), mute1-α5(IV), and mute2-α5(IV) chains was analyzed using molecular dynamics simulations over a period of 90 ns. (**B**) During a 90 ns period, the RMSD of the chains wild-α5(IV), mute1-α5(IV), and mute2-α5(IV) was analyzed. (**C**) The RMSF was calculated for every amino acid residue present in the wild-α5(IV), mute1-α5(IV), and mute2-α5(IV) chains over a time period ranging from 80 ns to 90 ns. (**D**) During a 100 ns simulation, the secondary structure counts of the wild-type α5(IV), mute1-α5(IV), and mute2-α5(IV) chains were determined

**Fig. 7:**
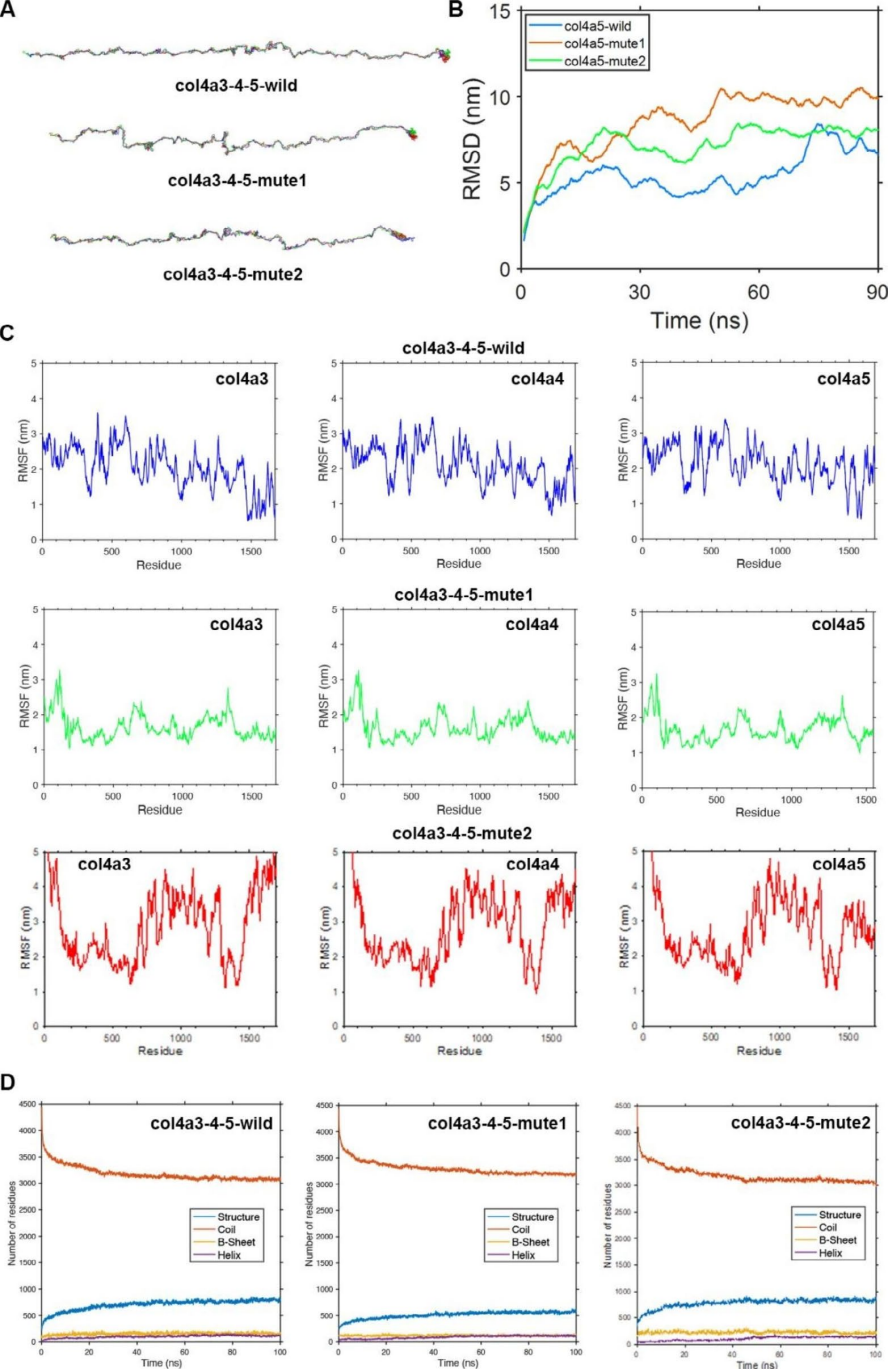
Protein molecular dynamics simulation of the α345(IV) trimer. (**A**) Molecular dynamics simulations were employed to investigate the conformation of the triple chains of wild-α345(IV), mute1-α345(IV), and mute2-α345(IV) over a period of 90 ns. (**B**) The RMSD of the triple chains comprising wild-α345(IV), mute1-α345(IV), and mute2-α345(IV) was evaluated over a period of 90 ns. (**C**) The RMSF was calculated for every amino acid residue within the triple chains of wild-α345(IV), mute1-α345(IV), and mute2-α345(IV) over a time period ranging from 80 ns to 90 ns. (**D**) During a 100 ns simulation, the secondary structure counts were determined for the triple chains of wild-α345(IV), mute1-α345(IV), and mute2-α345(IV)

Homology modeling was employed to generate the 3-D structure of the α5(IV) and α345(IV) trimer. The amino acid type was utilized to present the 3-D structure of the wild-α5(IV), mute1-α5(IV), and mute2-α5(IV) chains. Additionally, the ribbon type presentation was utilized to provide the 3-D structure of the wild-α345(IV), mute1-α345(IV), and mute2-α345(IV) trimers. The p.D1472Mfs mutation in mute1-α5(IV) was observed to cause aggregation, resulting in a reduction in the overall potential energy (Fig. 5A). The mute1-α5(IV) chain start to collapse compared with the wild-α5(IV) chain (Fig. 6A). Substitution of the wild-α5(IV) chain segment following amino acid residue 1472 with the ' MHHNAHREHFRSMKAFLSCMYKEIKEPTVKTWGRLAAAFVALVPCLSCSATSIMFA-TLLQEMTILTGSLPQSPCQ ' segment yielded a notable augmentation in the β-sheet and α-helix content of the mute1-α5(IV) chain. Specifically, the quantity of β-sheet structures increased from 10 to 100, while the number of α-helices rose from 42 to 90 (Fig. 6D). The trimer structure of mute1-α345(IV) formed after p.D1472Mfs did not collapse entirely, which is an intriguing observation (Fig. 7A). In contrast to wild-α345(IV), the mute1-α345(IV) trimer exhibited a decrease in the number of β-sheets and α-helices. The number of β-sheet structures was reduced from 160 to 120, while the number of α-helices was reduced from 100 to 80 (Fig. 7D).

The spatial structure of mute2-α5(IV), which was created using p.(P1432_G1433insDYFVEI), was found to be significantly different from that of the wild-α5(IV) (Fig. 5B). Following the incorporation of the DYFVEI segment into the wild-α5(IV) chain, there was a notable augmentation in the quantity of β-sheet and α-helix structures within the mute2-α5(IV) chain. More specifically, the number of β-sheet structures increased from 10 to 100, while the number of α-helix structures increased from 42 to 90 (Fig. 6A). The entire mute2-α5(IV) structure initiates collapse (Fig. 6D). The formation of the mute2-α345(IV) trimer occurs subsequent to the p.(P1432_G1433insDYFVEI) mutation, with the structure exhibiting partial collapse (Fig. 7A). Upon comparison of the alterations in secondary structure quantities between the mute2-α345(IV) and wild-α345(IV) chains, it was observed that the mute2-α345(IV) chain exhibited an increase in the number of β-sheet and α-helix structures. Specifically, the quantity of sheet structures increased from 160 to 230, while the quantity of α-helix structures increased from 100 to 120 (Fig. 7D).

### Protein molecular dynamics simulation of the α5(IV) and α345(IV) trimer

In order to investigate the geometric structure deviation of the wild-α5(IV), mute1-α5(IV), and mute2-α5(IV) chains, a full atom molecular dynamics simulation was employed to conduct a 100 ns kinetic study. The RMSD results indicate that the wild-α5(IV) chain reached the equilibrium structure in a shorter time frame (approximately 30 ns) compared to the mute1-α5(IV) and mute2-α5(IV) chains, which required a longer duration (around 80 ns) to attain the equilibrium structure (Fig. 6B). This phenomenon can be attributed to alterations in the amino acid composition of the mute1-α5(IV) and mute2-α5(IV) chains, as evidenced by the subsequent RMSF analysis.

The RMSF results showed that the mute1-α5(IV) chain had a considerable effect on the distance of certain amino acids in the chain, which in turn strongly influenced the conformational fluctuations of the chain. Inserting the amino acid sequence ‘MHHNAHREHFRSMKAFLSCMYKEIKEPTVKTWGRLAAAFVALVPCLSCSATSIMFATLLQEMTILTGSLPQSPCQ’ after amino acid 1472 leads to a notable increase in fluctuations in both the head and the 800–1000 segment of the protein. Additionally, the fluctuations in the 1400-tail segment also increase significantly (Fig. 6C). The results of the RMSF analysis indicate a significant increase in the fluctuation of the 1430–1600 residues in the mute2-α5(IV) chain, as well as the fluctuation of the 900–1150 residues, due to the insertion of the DYFVEI segment (Fig. 6C).

In order to investigate the geometric structure deviation of the triple chains wild-α345(IV), mute1-α345(IV), and mute2-α345(IV), a full atom molecular dynamics simulation was conducted for a duration of 100 ns. The RMSD results indicate that the wild-α345(IV) chain attained equilibrium structure in a shorter time frame of approximately 30 ns, whereas the mute1-α345(IV) and mute2-α345(IV) chains required a longer duration of approximately 50 ns to reach equilibrium structure (Fig. 7B). This phenomenon can be attributed to the alteration of the amino acids in mute1-α345(IV) and mute2-α345(IV), as evidenced by the subsequent RMSF analysis. The RMSF analysis revealed that the 1300–1400 segment of mute1-α345(IV) exhibits significantly higher fluctuations and stronger head swings compared to other segments (Fig. 7C). The analysis of root mean square fluctuation (RMSF) indicated a significant increase in the fluctuation of the head and end regions, as well as residues 700–1400, in the mute2-α345(IV) chain following the insertion of the DYFVEI segment (Fig. 7C).

## Discussion

The correlation between genotype and phenotype in XLAS is widely recognized [7, 8]. According to Jais *et al*, the presence of large deletions and nonsense mutations is associated with a 90% likelihood of developing end-stage renal disease (ESRD) by the age of 30, which is higher than the 70% risk associated with splice site mutations and the 50% risk associated with missense mutations [6]. Gross and colleagues categorized males with XLAS into three distinct groups, as delineated below [8]. The mean age of end-stage renal disease (ESRD) onset for large rearrangements, frame shift, nonsense, and splice donor site mutations was 19.8 ± 5.7 years. For non-glycine or 3’ glycine missense mutations, in-frame deletions/insertions, and splice acceptor site mutations, the mean ESRD age was 25.7 ± 7.2 years. Lastly, 5’ glycine substitutions exhibited an even later onset of ESRD at a mean age of 30.1 ± 7.2 years [8]. Bekheirnia and colleagues have documented that individuals with missense mutations experience the onset of end-stage renal disease at an average age of 37 years, while those with splice site mutations experience it at 28 years, and those with truncating mutations experience it at 25 years [7]. Gong *et al* conducted a thorough analysis of deletion mutations found in the *COL4A5* gene within the Chinese population with XLAS [9]. Patients with deletion mutations experienced symptom onset at an average age of 9.1 ± 6.0 years [9]. In their study, Gong et al found that approximately 50% of male participants with deletion mutations experienced hearing loss, while roughly 25% developed eye lesions [9]. In our investigation, we have discovered a previously unreported deletion variant, namely c.4414delG, located in exon 47 of the *COL4A5* gene, as observed in patient 1. Furthermore, his clinical phenotype serves as additional validation for previous studies conducted on this topic [10]. In a meta-analysis conducted by Gross et al on genotype-phenotype correlations in X-linked AS, three types of typical XLAS were identified: severe, moderate-severe, and moderate. Cases of severe ESRD are characterized by adolescent onset (20 + years), with 80% of individuals suffering from hearing loss and 40% experiencing ocular lesions. This condition is thought to be associated with frameshift, premature arrests, large rearrangements, as well as mutations in the donor splice sites and NC1 domain [8, 11, 12]. Our findings generally support this classification criterion. Specifically, our present study observed that the patient with the c.4414delG deletion variant progressed to ESRD at the age of 22 years and exhibited severe XLAS symptoms, such as hearing loss and ocular lesions. In accordance with the ACMG guidelines, we assessed the pathogenicity of the c.4414delG as follows. PVS1: loss of function is one of the causative mechanisms of the *COL4A5* gene, this variant is a frameshift variant that arises from a single base deletion. This leads to a premature termination of the protein after a 75 amino acid substitution, which may result in nonsense-mediated mRNA degradation.; PP4: the clinical phenotypes exhibited a high degree of consistency with the hereditary disease caused by abnormalities in the *COL4A5* gene; PM2_Supporting: the variant is infrequent and absent from the gnomAD database. The deletion of a single base caused by c.4414delG is considered to be pathogenic when combined with these results.

In a study by Kandai Nozu et al., it was found that 29% of men with XLAS exhibited less severe symptoms, such as milder proteinuria, later onset of ESRD, and reduced incidence of hearing loss. Interestingly, all of these individuals had nontruncating mutations. These findings suggest that in-frame mutations, even those derived from a splice site mutation, may lead to a milder phenotype [13]. Patient 2 in our study presented with persistent haematuria and mild proteinuria, but did not exhibit sensorineural deafness or ocular abnormalities. Notwithstanding the relatively benign phenotype observed in patient 2, electron microscopy revealed irregular thinning and thickening of the glomerular basement membrane (GBM) exhibiting a diffuse basket-weave pattern, which are characteristic abnormalities associated with AS. Therefore, a diagnosis of AS was made. In patient 2, we identified a variant in exon 47 of the *COL4A5* gene (c.4298-20T > A), which has not been previously reported in the gnomAD database and has not been experimentally verified. In order to ascertain the impact of this variant on the primary splicing site, a range of bioinformatics platforms were employed, namely dbscSNV_ADA, SpliceAI, varSEAK, and dbscSNV_RF. All platforms consistently suggested that the variant leads to unusual splicing in the *COL4A5* gene. We utilized an in vitro minigene splicing assay to identify any abnormal splicing due to a variant in the *COL4A5* gene [14, 15]. The results of our study demonstrate that the c.4298-20T > A variant preserves 18 base pairs originating from intron 46 of *COL4A5* transcripts, resulting in the incorporation of 6 amino acids following the amino acid located at position 1432 in α5(IV). Consequently, we conducted a reassessment of the pathogenicity of the c.4298-20T > A variant in accordance with the ACMG guidelines. PVS1_Moderate: the observed splicing region variant induces the formation of a novel splicing acceptor within intron 46, thereby causing an insertion of 18 base pairs (equivalent to 6 amino acids) at the N-terminus of exon 47 without altering the reading frame; PP4: the clinical phenotypes exhibited a high degree of consistency with the hereditary disease caused by abnormalities in the *COL4A5* gene; PP3: the bioinformatics software utilized predicted the potential impact of the variant on gene splicing; PS3_Moderate: the findings of the functional analysis indicate that the variant has an impact on gene splicing, leading to the retention of an intron spanning 18 base pairs; PM2_Supporting: the variant is infrequent and has not been incorporated into the gnomAD database. Upon consolidation of the findings, the base insertion resulting from the c.4298-20T > A mutation was deemed to be of probable pathogenic nature.

The human GBM is composed of the α3(IV), α4(IV), and α5(IV) chains of Col-IV, which all have a similar primary structure. This structure includes a 25-residue ‘7S’ domain at the amino terminus, a collagen domain consisting of around 1400 Gly-X-Y repeats, and a non-collagen domain of approximately 230 residues at the carboxyl terminus (NC1) [16]. The recognition site and nucleation center for the cross-linking of the three alpha chains is located at the NC1 structure at the C-terminus [17]. The recognition of NC1 structure between peptide chains is closely linked to two specific structures. The first is a β-hairpin module, which is made up of 13 amino acid residues. The second is a docking site, also known as the variable region 3 (VR3), which is composed of 15 amino acid residues and has a highly variable amino acid sequence [17]. The β-hairpin module and VR3 sites located at the C-terminus of the three peptide chains have the ability to recognize each other. Specifically, the β-hairpin module in each chain can bind to the VR3 site in the adjacent chain through disulfide and hydro-phobic bonds. This binding process ultimately leads to the intertwining of the collagen domains in all three peptide chains, forming a triple helix structure [17]. Disulfide bonds play a crucial role in stabilizing the triple helix structure of collagen. They can form between the collagen domains of each peptide chain, ensuring structural integrity and strength. When a pathogenic variant affects one of the three α chains in the encoding gene, it can cause the breakdown of the normally well-organized glomerular basement membrane (GBM). The aforementioned degradation involves the division of the lamina densa within the glomerular basement membrane, commonly referred to as the basket weave alteration. Such modifications may accelerate the progression of glomerular sclerosis, ultimately culminating in renal impairment. The etiology of XLAS is attributed to pathogenic variants in the protein-coding gene, which result in the α5(IV) chains. Following a mutation in the *COL4A5* gene, two scenarios may occur: 1) complete loss or truncation of the α5-chain protein product; 2) a full-length protein product with amino acid substitution or insertion. The former scenario is readily comprehensible, as an incomplete protein may not function normally, leading to disease. Furthermore, it is widely accepted that amino acid substitutions/insertions can result in local kinks or abnormal folding, disrupting the triple helix structure of collagen molecules. These abnormally folded collagen molecules are more susceptible to protease degradation, rendering them prone to degradation [18]. To clarify the relationship between genotype and phenotype in these two patients, we used molecular dynamics simulation to analyze the impact of the c.4414delG deletion variant and c.4298-20T > A splicing variant on the protein’s ability to form a triple helix [19].

The *COLA45* gene is comprised of 51 exons, with the sizes of exons within *COL4A5* (excluding 5’- and 3’- untranslated sequences) ranging from 27 to 213 bp. Exon 1 contains a total of 283 bp, consisting of 202 bp of a 5’-untranslated sequence and 81 bp of a translated sequence that encodes a 26-residue long signal peptide. Exon 2 encodes the non-collagenous amino-terminal end and two Gly-X-Y triplets, while exons 2–47 encode the collagenous domain. Exon 47 serves as a junction exon, encoding the carboxyl-ter-minal end of the collagenous domain and a portion of the non-collagenous domain. In our study, the patient 1 presented a deletion variant c.4414delG in exon 47 of the *COL4A5* gene. This deletion resulted in a change from aspartic acid to methionine in codon 1472 of the α5(IV) chain encoded by this gene. This change caused a shift in the code by 75 amino acids and ultimately led to an early stop codon. It is evident that the NC1 structure of p.D1472Mfs is no longer present and the key recognition site for polymerization with the other two strands has been lost, based on the coding region of exons 47–51 of the *COL4A5* gene. To examine the impact of p.D1472Mfs on α345(IV) trimerization, we utilized molecular dynamics analysis to simulate the conformational shift of mute1-α345(IV) following the *COL4A5* gene mutation and modeled the 3D structures of both wild-α345(IV) and mute1-α345(IV). Through our modeling approach, we observed the final changes in the structure of the mute1-α345(IV) trimer after incorporating mutation information. Despite the absence of the carboxyl terminus including NC1 in mute1-α5(IV), our trimerization modeling results demonstrate that the mute1-α345(IV) trimer can still be formed. This can be attributed to the binding between the collagen domains of the three peptide chains via disulfide bonds. Due to the severe damage of NC1 of mute1-α5(IV), the critical binding site between it and wild-α3(IV) and wild-α4(IV) is no longer present. As a result, the stability of the mute1-α345(IV) triple helix is reduced. In male patients with a deletion variant of the *COL4A5* gene, the α5(IV) chain was not expressed in vivo. We suggest that this lack of expression may be due to the eukaryotic protein quality control system removing the damaged α5(IV) chain. Although the structure of mute1-α345(IV) did not completely collapse, it was significantly shortened compared to wild-α345(IV). Likewise, the c.4298-20T > A variant led to the incorporation of six amino acids following the amino acid located at position 1432 within the wild-type α5(IV) protein. The insertion, DYFVEI, was situated at the C-terminal end of the α5(IV) chain, conserving 18 base pairs from intron 46 of *COL4A5* transcripts. In order to gain a more comprehensive understanding of the impact of this mutation, we performed full atom molecular dynamics simulations on the mute2-α5(IV) chain utilizing a kinetic simulation process lasting 100 ns. The molecular dynamics findings suggest that the c.4298-20T > A variant has an impact on the stability of the α5(IV) chain’s tail and middle regions. To conduct a more in-depth analysis, a 100 ns kinetic simulation process was performed utilizing full atom molecular dynamics simulations on the mute2-α345(IV) trimer. The results obtained from the molecular dynamics simulations revealed that the c.4298-20T > A variant had a significant impact on the stability of not only the tail of the α345(IV) trimer, but also its head and middle regions. The results indicate that the mute2-α345(IV) trimer, which arises from an intron 46 splicing variant in the *COL4A5* gene, experiences notable structural modifications, albeit without complete collapse.

In this study, we successfully modelled the triple helix structure of mute-α345(IV) using a molecular dynamics approach. We also monitored the variation of the trimer using variation information. Upon comparing the RMSF results of mute1-α345(IV) trimer and mute2-α345(IV) trimer, it is evident that the fluctuations in the tails of the wild-α3(IV) and wild-α4(IV) chains in mute1-α345(IV) trimer are considerably weaker than that of mute2-α345(IV) trimer. This is likely due to the fact that the NCI regions of the wild-α3(IV) and wild-α4(IV) chains are unable to complete normal trimerization after a severe deletion of the NC1 region of the mute1-α5(IV) chain. As a result, the RMSF values of the NC1 region of both wild-type chains are significantly reduced. In contrast, despite the insertion of additional amino acid residues, the NCI region of the mute2-α5(IV) chain of the mute2-α345(IV) trimer remains intact. Therefore, the NC1 of the wild-α3(IV) chain and wild-α4(IV) chain of the mute2-α345(IV) trimer are still active and can complete trimerisation with the mute2-α5(IV) chain. This is supported by significant fluctuations in the RMSF values of these two wild-type chains. Based on the clinical phenotype of the two patients discussed in this study, patient 1 exhibited a more severe condition than patient 2. We suggest using molecular dynamics simulations of the mute-α345(IV) trimer to predict the severity of a patient’s clinical phenotype. This can be done by analyzing fluctuations in the RMSF values of the NC1 region of the wild-α(IV) chain of the trimer. At this stage, it is still uncertain whether molecular dynamics simulations can accurately reflect the clinical severity of different pathogenic variants of AS. Our research is in its early stages and more data is needed to draw definitive conclusions. However, our laboratory is actively working on this topic.

At this stage, more than 8,000 single-gene genetic diseases have been identified, which usually have a low incidence but a wide variety, resulting in an overall high incidence of 1 in 100. most single-gene genetic diseases have an early onset, are highly dangerous, can be teratogenic, disabling, or severe enough to cause death, and lack effective treatments. Studies have shown that in addition to the relatively common dominant genetic disorders represented by AS, the average healthy person carries 2.8 disease-causing variants of severe recessive monogenic genetic disorders, meaning that phenotypically healthy couples may carry disease-causing variants of the same gene, resulting in a 25% probability of disease in their offspring. Therefore, a three-tier approach to prevention, starting with pre-conception carrier screening, prenatal screening and screening for newborn diseases, is the main focus of birth defect prevention and control. The identification of cffDNA in maternal plasma in recent times has facilitated the emergence of novel clinical applications that depend on the scrutiny of fetal genetic material. The cffDNA component accounts for roughly 3–13% of maternal free DNA and originates from placental cells that have undergone apoptosis, subsequently entering the maternal circulation. With the rapid development of sequencing technologies, the identification of cffDNA in the maternal circulation has enabled the analysis of circulating cffDNA with considerable sensitivity and specificity. Non-invasive prenatal testing for aneuploidy screening of pregnant women is now established worldwide [20]. A highly sensitive and specific genome-wide amplification method for cffDNA purification and enrichment was developed and validated by Alyafee et al. This method has the potential to facilitate non-invasive screening for monogenic diseases at the prenatal stage, particularly for high-risk pregnant women with children affected by family-specific AR disorders [21]. Furthermore, for monogenic diseases, genetic screening/diagnosis is the best strategy to prevent such diseases for which no treatment is currently available. Recently, Alyafee et al. reported the availability of a high throughput next generation sequencing (NGS) based aneuploidy pre-implantation genetic testing technology. This technology reduces public health and family costs for families with a history of genetic disorders by blocking monogenic diseases ahead of embryo transfer and completion of pregnancy [22]. In addition, the Newborn Screening Programme (NBS) is now recognized worldwide as a highly successful public health programme for health promotion and disease prevention, but the number of diseases screened varies from country to country, the reason for this variation lies in the question of which and how many diseases should be included in the newborn screening panel, and reporting cases associated with these genes will help to determine genotype-phenotype correlations and facilitate future clinical trials [23]. In conclusion, appropriate genetic counselling for affected families is essential in the presence of monogenic genetic disorders.

## Conclusions

To conclude, this study reports the identification of two variants in the *COL4A5* gene, namely a deletion variant c.4414delG and a splice variant c.4298-20T > A, in two patients with XLAS. By conducting histopathological examination of the kidney, the study provides in vivo evidence for the pathogenicity of these variants and expands the spectrum of variants associated with XLAS. The findings of this study contribute to a better understanding of the molecular pathogenesis of AS. Both variants have been uploaded to the ClinVar database with accession numbers VCV001711180.1 and VCV000974467.3.

### Electronic supplementary material

Below is the link to the electronic supplementary material.

Supplementary Material 1

Supplementary Material 2

## Data Availability

The datasets utilized in this study are available on ClinVar (https://www.ncbi.nlm.nih.gov/clinvar/RCV001281232.2/). The data presented in the study are deposited in the Genome Sequence Archive in National Genomics Data Center, accession number HRA003934.
