# Supplementary figures and images for: Genetic and molecular dynamics analysis of two variants of the COL4A5 gene causing Alport syndrome
Source: BMC Med Genomics. 2023 Aug 18;16:192. doi: 10.1186/s12920-023-01623-7 (PMC10436629; doi:10.1186/s12920-023-01623-7)

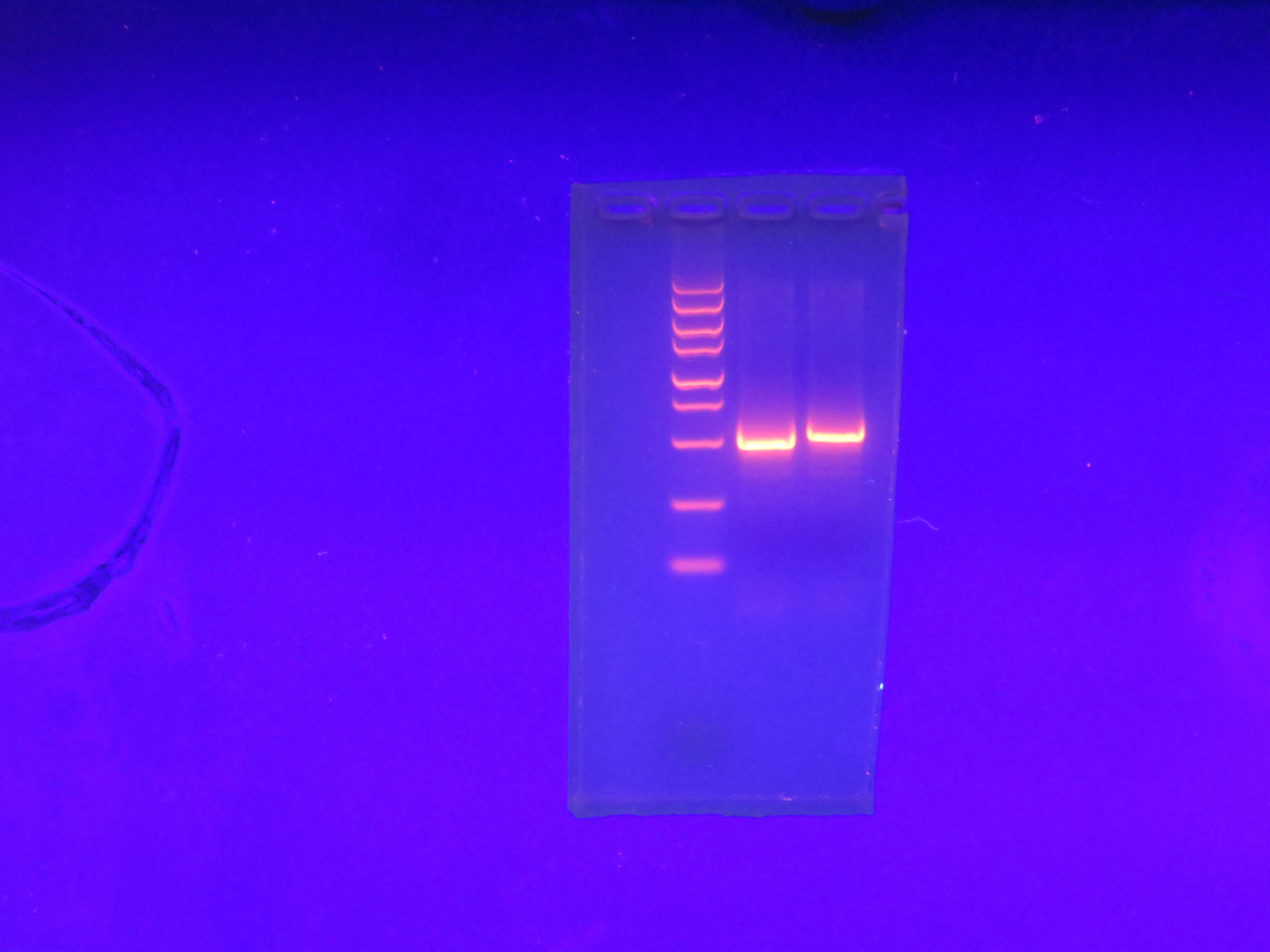

Supplement: Supplementary file 1 — Supplementary Material 1 [file 12920_2023_1623_MOESM1_ESM.jpg]

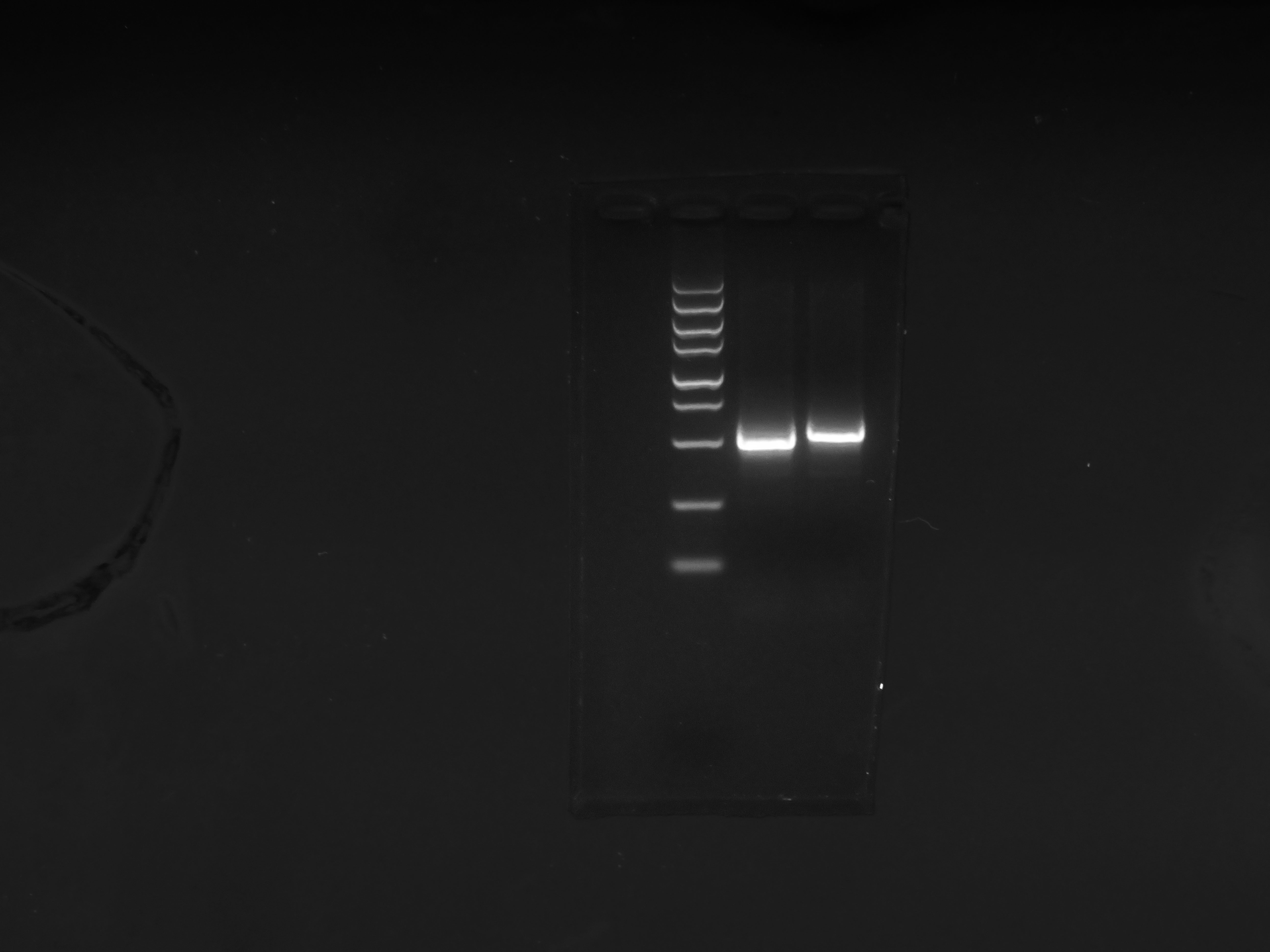

Supplement: Supplementary file 2 — Supplementary Material 2 [file 12920_2023_1623_MOESM2_ESM.jpg]
